# Genome-Wide Identification and Expression Analysis of the 4-Coumarate: CoA Ligase Gene Family in *Solanum tuberosum*

**DOI:** 10.3390/ijms24021642

**Published:** 2023-01-13

**Authors:** Tengkun Nie, Xinxin Sun, Shenglan Wang, Dongdong Wang, Yamei Ren, Qin Chen

**Affiliations:** 1 State Key Laboratory of Crop Stress Biology for Arid Areas, College of Food Science and Engineering, Northwest A&F University, Yangling 712100, China; 2 College of Agronomy, Northwest A&F University, Yangling 712100, China

**Keywords:** potato, 4-coumarate: CoA ligase (4CL), abiotic stress, expression analysis, gene family

## Abstract

4-coumarate: CoA ligase (4CL) is not only involved in the biosynthetic processes of flavonoids and lignin in plants but is also closely related to plant tolerance to abiotic stress. UV irradiation can activate the expression of *4CL* genes in plants, and the expression of *4CL* genes changed significantly in response to different phytohormone treatments. Although the *4CL* gene has been cloned in potatoes, there have been fewer related studies of the 4CL gene family on the potato genome-wide scale. In this study, a total of 10 potato *4CL* genes were identified in the potato whole genome. Through multiple sequence alignment, phylogenetic analysis as well as gene structure analysis indicated that the potato 4CL gene family could be divided into two subgroups. Combined with promoter cis-acting element analysis, transcriptome data, and RT-qPCR results indicated that potato 4CL gene family was involved in potato response to white light, UV irradiation, ABA treatment, MeJA treatment, and PEG simulated drought stress. Abiotic stresses such as UV, ABA, MeJA, and PEG could promote the up-regulated expression of *St4CL6* and *St4CL8* but inhibits the expression of *St4CL5*. The above results will increase our understanding of the evolution and expression regulation of the potato 4CL gene family and provide reference value for further research on the molecular biological mechanism of 4CL participating in response to diverse environmental signals in potatoes.

## 1. Introduction

4-coumarate: CoA ligase (4CL; EC 6.2.1.12) is a key enzyme in the phenylalanine metabolic pathway [1]. Studies have shown that 4CL is mainly specific for 4-coumaric acid and cinnamic acids and other derivatives of cinnamic acid [2]. 4CL catalyzes the reaction of 4-coumarate, coenzyme A and ATP to produce 4-Coumaroyl-CoA along with AMP and diphosphate [3]. 4-Coumaroyl-CoA is further involved in the biosynthesis of secondary metabolites such as flavonoids and lignin [4]. In the 1970s, p-coumarate: CoA ligase was isolated from soybean and the xylem of *Forsythia suspensa*, respectively [5,6]. This was followed by studies showing a significant increase in the content of flavonoids in overexpressing *Rehmannia glutinosa 4CL2* transgenic lines [7]. Meanwhile, lignin content was increased in transgenic tobacco of overexpression *Camellia sinensis 4CL1* gene [8]. Studies on *4CL* gene mutants of Arabidopsis showed that lignin content was reduced in *At4CL1* mutants, whereas *At4CL1 At4CL2* double and *At4CL1 At4CL2 At4CL3* triple mutants not only had reduced lignin content and exhibited the characteristic of plant dwarfism after flowering [4]. The transgenic rice with suppressed *Os4CL3* expression similarly showed reduced lignin and plant height dwarf [9]. The above studies indicate that *4CL* genes play important roles in the process of plant lignin and flavonoids accumulation.

4CL gene families have been found in plant genomes such as Arabidopsis, rice, and pomegranate [10,11]. The 4CL gene family in a variety of plants contains both AMP-binding enzyme domain (AMP-binding; PF00501) and AMP-binding enzyme C-terminal domain (AMP-binding_C; PF13193) [12]. AMP-binding is the core functional region of the 4CL gene family, which facilitates covalent binding between reaction substrates through ATP dependence [13]. The amino acid functional region of AMP-binding is rich in Ser/Thr/Gly-rich and contains a highly conserved Pro-Lys-Gly amino acid triplet [14]. AMP-binding_C generally contains a smaller number of amino acids, which are generally located at the C-terminus of the polypeptide chain [15]. AMP-binding_C is centered on a Beta turn, and both sides of the Beta turn are Alpha helices [16]. Since AMP-binding and AMP-binding_C are required for 4CL to function catalytically, the inclusion of both AMP-binding and AMP-binding_C is also included as an important criterion to judge whether 4CL is or not [17].

The *4CL* genes also participated in the plant response to multiple abiotic stresses [18]. Ultraviolet (UV) irradiation is one type of light stress (photo-stress), and high-intensity UV irradiation shortens plant internodes, impedes pollen formation, and inhibits plant growth and development [19]. Both the *Os4CL2* gene in rice and the *At4CL3* gene in Arabidopsis increased significantly under UV irradiation [11,20]. Thus, 4CL was involved in plant response to light stress from UV irradiation. The overexpression of *Fraxinus mandshurica 4CL* gene in transgenic tobacco significantly increased lignin content and thus improved the drought resistance of transgenic tobacco [21]. The expression of *4CL* in *Fagopyrum tataricum* could be inhibited by abscisic acid (ABA) treatment [22]. Meanwhile, *4CL* can also respond to Methyl jasmonate (MeJA) treatment in different plants [23]. Further studies showed that 4CL family genes from a variety of plants such as Arabidopsis and rice could be classified into two types with differences in structure and function, one of which is involved in lignin synthesis and the other in the biosynthesis of phenylpropanoids other than lignin [24]. *4CL* genes also have important biological functions in potato (*Solanum tuberosum*), and studies have shown that up-regulated expression of *4CL* is one of the effective ways for a potato to defend against *Phytophthora infestans* [25]. *4CL* genes of potato (St4CLs) have high amino acid sequence homology with *Arabidopsis thaliana 4CL1* and *4CL2* [11]. 4CL is an important functional protein responsible for the biosynthesis of flavonoids and lignin in potatoes; however, there has been no systematic study on the *St4CLs* [26]. At present, there have been no related research based on genomic data to identify members of the 4CL gene family in potato and to investigate the relationship of the potato 4CL gene family evolution as well as expression patterns.

4CL plays an important role in both plant secondary metabolite synthesis and tolerance to abiotic stress [27]. Therefore, the study of St4CL family genes will not only help to elevate the contents of nutrients such as flavonoids and lignin in potato but is particularly important for enhancing the ability of potatoes to withstand abiotic stresses. With the completion of a fine map of potato genome constructed by applying Nanopore Technologies long reads coupled with High-throughput/resolution chromosome conformation capture, it provided a possibility to deeply investigate the potato *4CL* homologous gene [28]. This study applied the latest potato genome sequencing results, combined with bioinformatics methods, to identify 4CL gene family members in potatoes. Meanwhile, the expression patterns of *4CL* genes under white light, UV, PEG, ABA, and MeJA treatments were investigated by RT-qPCR. The above studies provide important clues for further revealing the biological functions of *St4CLs*.

## 2. Results

### 2.1. Identification of 4CL Gene Family Members in Potato

The 4CL protein sequence of *Arabidopsis thaliana* and the protein sequence of cloned potato 4CL were used to Blast in the potato genome *database* [29]. We only retained potato genes with E-value less than or equal to 1 × e^−10^ in Blast results as candidate genes for the St4CL gene family [30]. The results 51 genes were derived after Blast application of the Arabidopsis 4CL protein sequence, and 47 genes were derived after Blast application of the potato 4CL protein sequence. After merging and deleting duplicated genes from the above two Blast results, 51 candidate genes of potato *4CLs* were obtained. We calculated the Blast result through HUMMER software and looked it up in the SMART database, obtaining 11 genes containing PF00501 and PF13193 domains while not containing other domains. Soltu.DM.03G000120 nitrogen terminus lacks 56 amino acids by sequence alignment and KEGG results indicated that the protein is protoporphyrinogen oxidase (EC 1.3.3.4), so it does not belong to the 4CL gene family [31]. Eventually, 10 potato 4CL family genes were identified from the potato genome (Table 1). To preliminarily analyze the protein functions of the St4CL family genes, a ExPASy-ProtParam tool was applied to analyze the physicochemical properties of St4CL proteins.

In this study, amino acid numbers, protein molecular weights (MW), and isoelectric points (pI) of each gene in the St4CL family were analyzed, along with predictions of subcellular localization (Table 1). The results indicated that the St4CL proteins length ranged from 545 to 616 amino acids, from 59,636.17 to 68,009.17 in molecular weight, and from 5.36 to 8.21 in pI. St4CL proteins were predicted to be distributed mainly in the bio-membrane systems, where they were detected at both the plasma and organelle membranes.

### 2.2. Phylogenetic Analysis of Potato 4CL Gene Family

**Figure 1:**
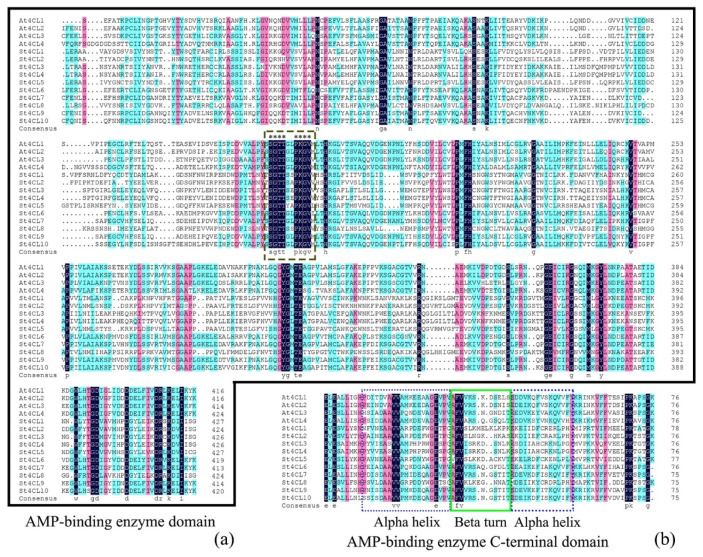
4CL gene family domains in Arabidopsis and potato were used to multiple sequence alignment. (**a**) The AMP-binding enzyme domain amino acid sequences were aligned. The dashed boxes were conserved amino acid sequences. “*” were conserved amino acids in the AMP-binding enzyme domain. (**b**) The AMP-binding enzyme C-terminal domain amino acid sequences were aligned. The solid boxes were amino acid sequences corresponding to the Beta turn, and dashed boxes were amino acid sequences corresponding to the Alpha helices.

To perform phylogenetic analysis of St4CL gene family, multiple sequence alignments of conserved domains of 4CL protein sequences in potato and Arabidopsis were first performed. The conserved domains amino acid comparison analysis indicated the inclusion of SGTT-PKGV (Ser Gly Thr Thr- Pro Lys Gly Val) amino acid sequence in both Arabidopsis and potato AMP-binding enzyme domain (Figure 1a). The SGTT region was a Ser(S)/Thr(T)/Gly(G) amino acid rich region, and immediately adjacent to it was a region containing a conserved Pro(P)-Lys(K)-Gly(G) amino acid triplet. These conserved amino acid sequences were fundamental to the maintenance of AMP-binding enzyme domain (AMP-binding) function [13,14]. In addition, valine (Val, V) was also highly conserved in AMP-binding of 4CL proteins in Arabidopsis and potato. At the same time, we also performed analyses of the protein secondary structures of potato and Arabidopsis AMP-binding enzyme C-terminal domain (Figure 1b). The results indicated the inclusion of a central Beta turn flanked by Alpha helices in both potato and Arabidopsis AMP-binding enzyme C-terminal domain (AMP-binding_C) [16]. The AMP-binding and AMP-binding_C are structurally conserved in Arabidopsis and potato, and both domains are indispensable for St4CLs to perform its normal biological functions.

**Figure 2:**
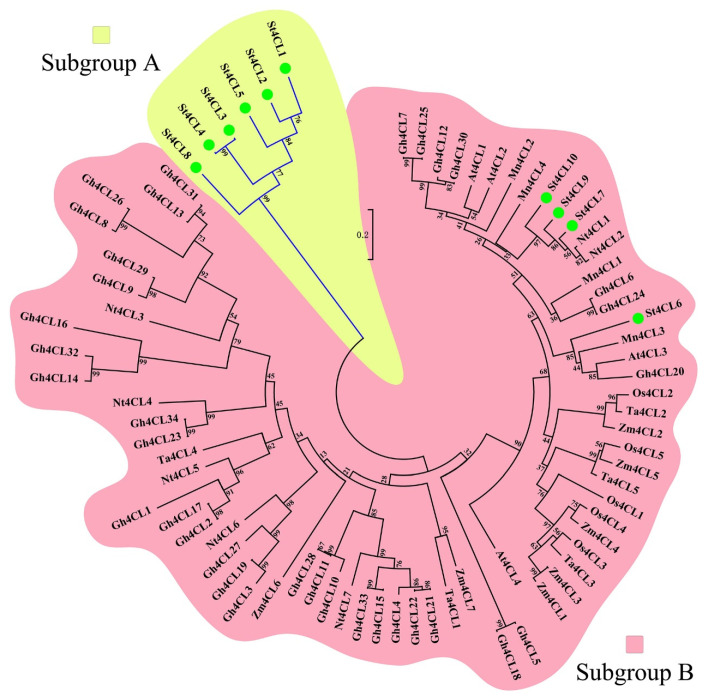
Phylogenetic analysis of *4CL* genes in plants. Phylogenetic analysis was performed by the neighbor-joining (NJ) method using MEGA10 software with a bootstrap test of 1000 replicates. The Yellow region is the branch that subgroup A contains, and the pink region is the branch that subgroup B contains.

A phylogenetic tree comprising potato (*Solanum tuberosum*), *Arabidopsis thaliana*, rice (*Oryza sativa*), tobacco (*Nicotiana tabacum*), cotton (*Gossypium hirsutum*), mulberry (*Morus notabilis*), maize (*Zea mays*), wheat (*Triticum aestivum*) was constructed by the neighbor-joining (NJ) method and used to study the phylogenetic relationships of 4CL gene family (Supplementary Table S1). Based on the bootstrap test results, the potato *4CL* genes were divided into two subgroups, namely subgroup A and subgroup B (Figure 2). Six *St4CL* genes are included in subgroup A, and *4CL* genes of other species are included in subgroup B along with four *St4CLs*. It follows that *St4CLs* in subgroup A, although similarly possessing conserved domains, from the perspective of phylogeny, have diverged from other plant *4CL* genes. In subgroup B, the *4CL* genes in monocots (rice, maize, wheat) are more closely related to those in dicotyledons (potato, *Arabidopsis thaliana*, tobacco, cotton, mulberry). *St4CLs* in subgroup B did not diverge from other plant *4CL* genes.

### 2.3. Analysis of the Gene Structures of the Potato 4CL Gene Family

**Figure 3:**
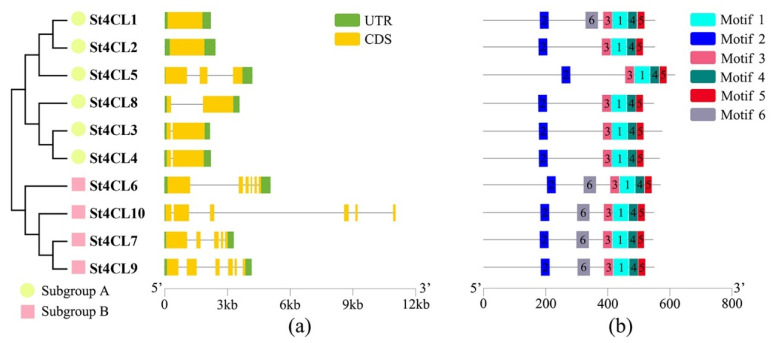
Gene structure analysis and identification of conserved motifs. (**a**) The structural analysis of St4CL gene family members. Green color bars are the UTR regions, yellow color bars are the CDS regions, and the solid black lines represent introns. (**b**) Analysis of conserved motifs in the St4CL gene family.

To further investigate the phylogenetic relationships of St4CL gene family and analyze the structural differences of *St4CL* genes, we performed analyses of the gene structures of St4CL gene family as well as the motifs of potato 4CL proteins. The *St4CLs* in subgroup A universally contained one to three exons, with no introns present in the *St4CL1* and *St4CL2* (Figure 3a). Whereas in subgroup B the genes contained more introns. *St4CLs* in subgroup B universally contained 5 to 6 exons and 4 to 5 introns. Therefore, the gene structures of the same subgroups were similar, but there were significant differences in gene structures among the different subgroups.

In total, six conserved motifs were recognized among the St4CL gene family (Figure 3b). All St4CL proteins contained motif 1, motif 2, motif 3, motif 4 and motif 5; however, motif 6 was only present in St4CL1, St4CL6, St4CL7, St4CL9 and St4CL10. The number of amino acids included by these motifs varied from 23 to 50 (Table 2). Motif 2 contains the SGTT-PKGV conserved amino acid region, and thus motif 2 should be a critical part in the AMP-binding enzyme domain. Only St4CL1 in subgroup A had motif 6, and all St4CLs in subgroup B contained motif 6. From this, it was inferred that the variation of amino acids in motif 6 was an important cause for the phylogenetic divergence of the potato 4CL gene family, which in turn differentiated into two subgroups.

### 2.4. Tandem Duplication of St4CL and Collinearity Analysis of the 4CL Gene Family among Different Species

**Figure 4:**
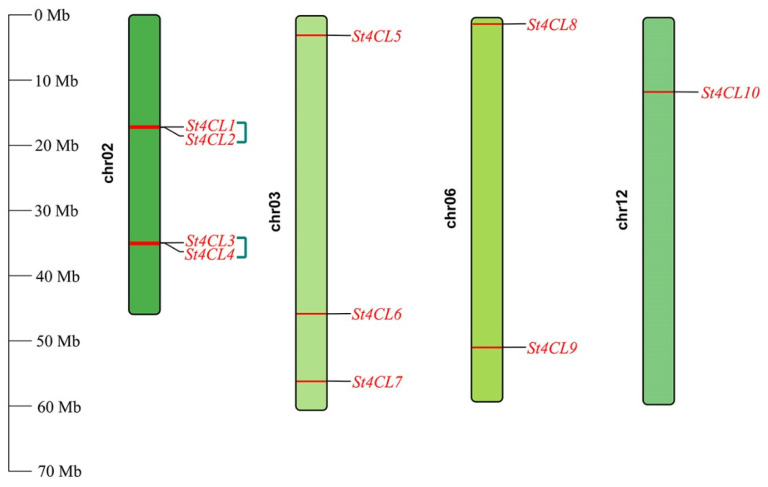
Distribution of the St4CL gene family on potato chromosomes. The two genes connected by green lines were tandem duplication genes.

We have undertaken a tandem duplication analysis of the potato 4CL gene family in order to investigate how the *St4CLs* might be expanding in the potato genome. Firstly, we obtained the distribution of the *St4CLs* on chromosomes (Figure 4). The St4CL genes were found exclusively on potato chromosomes 2 (chr02), chromosomes 3 (chr03), chromosomes 6 (chr06), and chromosomes 12 (chr12). The chr02 was exceptional compared to other chromosomes, with four *St4CL* genes distributed on chr02. According to the principle of tandem duplication gene identification [30], four *St4CL* genes on chr02 were two groups of *St4CL* tandem duplication genes (Figure 4). They were *St4CL1* and *St4CL2* as a group of tandem duplication genes, *St4CL3* and *St4CL4* as a group of tandem duplication genes, respectively, whereas no cases of *St4CLs* tandem duplication were found on other chromosomes.

**Figure 5:**
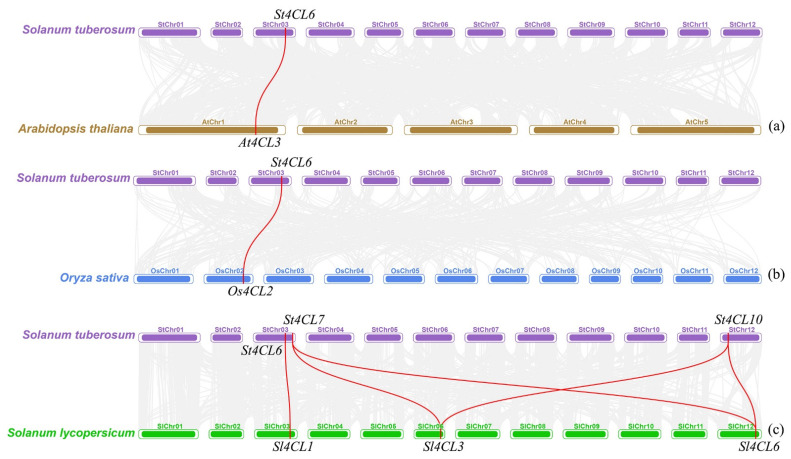
*4CL* genes in rice (*Oryza sativa*), potato (*Solanum tuberosum*), *Arabidopsis thaliana* and tomato (*Solanum lycopersicum*) were analyzed for collinearity. (**a**) *4CL* genes in potato and Arabidopsis were analyzed for collinearity. (**b**) *4CL* genes in potato and rice were analyzed for collinearity. (**c**) *4CL* genes in potato and tomato were analyzed for collinearity. Gray lines represent regions where collinearity exists between rice, potato, Arabidopsis and tomato. The red lines were used to connect genes for which collinearity existed between rice, potato, Arabidopsis, and tomato.

To investigate how *4CL* genes may be expanded across species genomes, we performed collinearity analysis of the 4CL gene family in the Arabidopsis, potato, rice and tomato (*Solanum lycopersicum*) genomes [32]. There was collinearity between potato *St4CL6* and Arabidopsis *At4CL3* (Figure 5a), while *St4CL6* was also collinear with rice *Os4CL2* (Figure 5b). The *4CL* in potato is collinear with that in dicotyledon Arabidopsis and monocotyledon rice, which indicates that the 4CL gene family in potato does not exist in isolation. The presence of three genes in tomatoes (Supplementary Table S1), which is the same solanaceous plant as potato, showed collinearity with *St4CL6*, *St4CL7*, and *St4CL10* in potatoes (Figure 5c). This illustrated that the potato 4CL gene family was more closely related to the tomato 4CL gene family during evolution compared to Arabidopsis and rice. In the St4CL gene family, *St4CL6* has collinearity with 4CL gene in rice, Arabidopsis, and tomato, so *St4CL6* may play an important role in the evolution of the St4CL gene family of potato genome and in the process of gene expansion of the St4CL gene family.

### 2.5. Analysis of Promoter Cis-Acting Elements in the St4CL Gene Family

**Figure 6:**
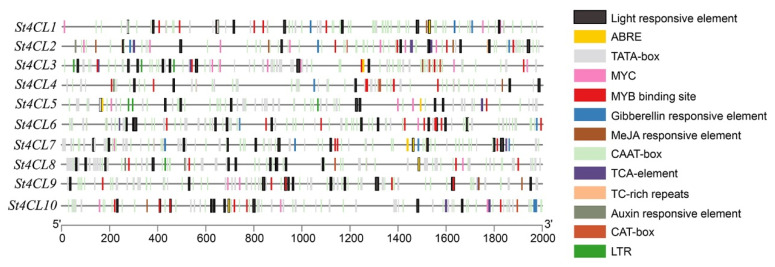
Analysis of cis-acting elements in the *St4CL* gene promoters. The genomic sequence 2000 bp upstream of the start codon was selected as the promoter for analysis.

The cis-acting elements in the promoters are important molecular switches for plants to trigger changes of gene expression in response to abiotic stresses [33]. In order to further research the transcriptional regulation mechanism of St4CL gene family under abiotic stress, this study analyzed the promoter cis-acting elements of St4CL gene family members. Through in-depth analysis of the 2kb genome sequence upstream of the starting codon, the promoter of the St4CL gene family contained 13 cis-acting elements (Figure 6). All *St4CL* promoter sequences contained light responsive element, MYB binding site that responded to various abiotic stress signals, TATA-box and CAAT-box that were common cis-acting elements in promoter sequences. Except for *St4CL6*, the rest of *St4CL* promoters contained ABRE cis-acting elements in response to ABA treatment [34]. Moreover, analysis of the St4CL *promoter* revealed that *St4CLs* were also involved in signal transduction in response to gibberellin, MeJA, auxin, and have MYC transcription factor binding sites. Therefore, *St4CL* genes were involved in multiple transcriptional regulatory processes in potatoes.

### 2.6. Expression Regulation Analysis and Protein Interaction Network Analysis

**Figure 7:**
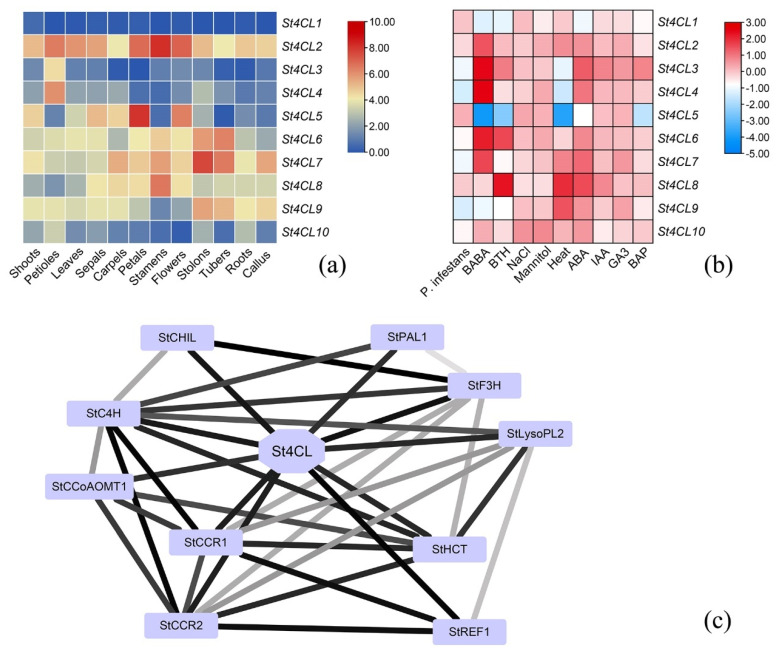
Analysis of *St4CLs* expression based on transcriptional data as well as protein interaction network analysis of St4CL gene family. (**a**) Gene expression profiles of *St4CLs* in different tissues of potato. (**b**) *St4CLs* expression changed under different adversity stresses. (**c**) The protein–protein interaction network analysis of the St4CL gene family. Lines represent direct or indirect functional relevance of the two proteins.

To further understand the transcriptional regulatory processes that the St4CL gene family involved in potato, we investigated the expression regulation of *St4CL* in different tissues as well as in different treatments by analyzing the transcripts per million (TPM) values of *St4CLs* from potato RNA-seq data [28,35]. The results showed that the expression of *St4CL1* was lower in various tissues of potato, in contrast to *St4CL2*, which was expressed in higher amounts in potato (Figure 7a and Supplementary Table S2). The *St4CL5* was mainly expressed in potato petals, and expression of the *St4CL5* could also be detected in roots. *St4CL6*, *St4CL7*, and *St4CL8* were highly expressed in all potato organs examined, especially *St4CL6* and *St4CL7* were mainly expressed in stolons and tubers of potatoes. We analyzed the expression pattern of *St4CL* genes by different treatments (Figure 7b and Supplementary Table S3). After *Phytophthora infestans* (*P.infestans*) infection, the expression of *St4CL4* and *St4CL9* was suppressed, and the expression of *St4CL1* and *St4CL5*, and *St4CL8* increased. *St4CL5* showed decreased expression under abiotic stress in β-aminobutyric acid (BABA), benzothiadiazole (BTH), abscisic acid (ABA), and heat, whereas the other St4CL family genes mostly showed increased expression. The expressions of St4CL family all appeared to rise under the treatment of NaCl, mannitol, auxin (IAA) and gibberellin (GA3).

The analysis of protein–protein interaction network can deeply explain the metabolic regulation process of St4CL gene family involved in functional protein association network [36]. This protein interaction regulation is not only a direct physical interaction, but more an indirect correlation of protein function [37]. St4CL gene family was found to interact with phenylalanine ammonia (StPAL), coniferyl-aldehyde dehydrogenase (StREF1), caffeoyl-shikimate esterase (StLysopl2), Cinnamoyl-CoA reductase 1 (StCCR1) and Cinnamoyl-CoA reductase 2 (StCCR2), which were key enzymes in the synthesis of potato lignin, as well as flavanone 3-hydroxylase (StF3H) and chalcone isomerase (StCHIL), which were involved in flavonoids biosynthesis (Figure 7c). This illustrated that the potato 4CL gene family was not only a key enzyme for the synthesis of lignin but was also involved in the regulation of flavonoids biosynthesis. In addition, St4CL gene family proteins interacted with hydroxycinnamoyl transferase (StHCT), caffeoyl coenzyme A methyltransferase (StCCoAOMT1) and cinnamic acid 4-hydroxylase (StC4H), so St4CL gene family is also involved in stilbene, diarylheptanoid, and gingerol biosynthesis as well as ubiquinone and other terpenoid-quinone biosynthesis processes [38].

### 2.7. Expression of St4CLs under Different Treatments and St4CL5 Subcellular Localization 

**Figure 8:**
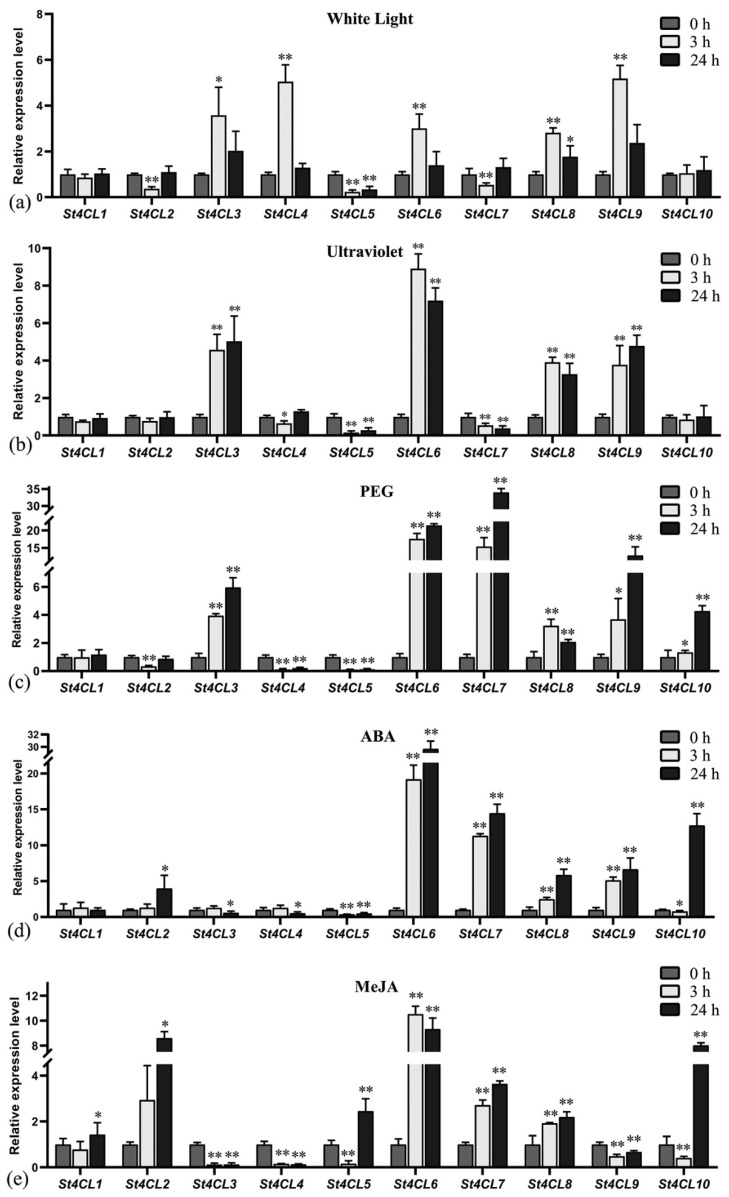
*St4CL* genes expression changed in response to different treatments. (**a**) Relative expression levels of *St4CLs* under constant white light treatment. (**b**) Relative expression levels of *St4CLs* under ultraviolet radiation. (**c**) Relative expression levels of *St4CLs* upon PEG simulated drought stress. (**d**) Relative expression levels of *St4CLs* under ABA treatment. (**e**) Relative expression levels of *St4CLs* under MeJA treatment. The data are shown as mean values ± SD (n = 3). (* *t*-test *p*-value < 0.05, ** *t*-test *p*-value < 0.01).

Combined with the promoter analysis results as well as potato RNA-seq results, St4CL gene family was involved in multiple environmental signal responses and participates in multiple phytohormone responses. To advance the determination of the expression pattern of the St4CL gene family under abiotic stress, the expression of the St4CL gene family under continued white light, UV, PEG, ABA, and MeJA treatment was examined by RT-qPCR. *St4CLs* showed significant changes in expression when potatoes were treated with white light for 3 h. *St4CL3*, *St4CL4*, *St4CL6*, *St4CL8*, and *St4CL9*, all of which showed a significant increase in expression after 3 h of white light treatment, and the expression of these genes decreased gradually with continued white light exposure (Figure 8a). This illustrated that the St4CL gene family was involved in light signal responses. To further identify the key genes involved in photo-stress in the St4CL gene family, we applied UV light as photo-stress irradiating potato seedlings [39]. When UV was continuously irradiated for 3 h and 24 h, *St4CL3*, *St4CL6*, *St4CL8*, and *St4CL9* showed significant up regulation in gene expression (Figure 8b). The expression of *St4CL3*, *St4CL6*, *St4CL7*, *St4CL8*, *St4CL9*, and *St4CL10* increased significantly after PEG treatment, with the expression of *St4CL7* at 24 h of PEG treatment reaching 34-fold of that at untreated (Figure 8c). *St4CL2*, *St4CL6*, *St4CL7*, *St4CL8*, *St4CL9*, *St4CL10* showed significant up regulation of gene expression after ABA treatment (Figure 8d). The expression of *St4CL2*, *St4CL6*, *St4CL7* and St4CL8 was similarly up-regulated after MeJA treatment (Figure 8e).

Decreased gene expression was also seen in the St4CL gene family in different treatments. *St4CL2* and *St4CL7* showed a significant decrease in expression at 3 h of white light treatment, but the expression of these genes became indistinguishable when subjected to continuous light for 24 h compared with that without white light treatment (Figure 8a). *St4CL5* appeared as a negatively regulated gene in response to white light treatment, with expression 0.24-fold and 0.34-fold of control (light treatment for 0 h) after 3 h and 24 h of continuous white light treatment, respectively. The gene expression of both *St4CL5* and *St4CL7* appeared to significant decrease after UV irradiation, in which *St4CL5* was only 0.16-fold of the control after 3 h of UV treatment (Figure 8b). Both *St4CL4* and *St4CL5* showed significant decreases in expression when treated with PEG, and similarly *St4CL4* and *St4CL5* showed decreased gene expression under ABA treatment (Figure 8c,d). When treated with MeJA, gene expression of *St4CL3*, *St4CL4*, and *St4CL9* decreased significantly, which indicated that *St4CL3*, *St4CL4*, and St4CL9 were involved in negative regulation of MeJA signal response in potatoes (Figure 8e).

**Figure 9:**
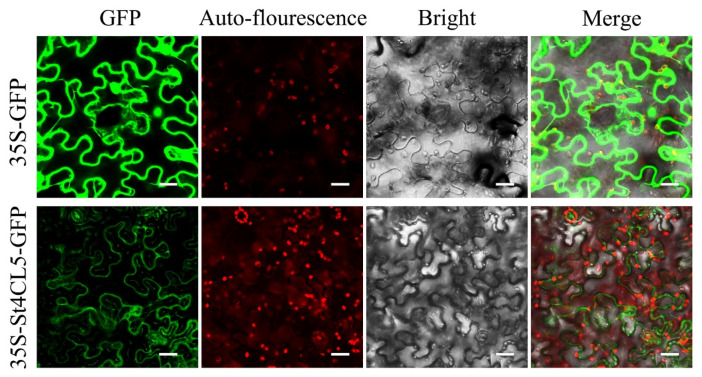
Subcellular localization of St4CL5 protein in potato. 35S-GFP as the experimental control was transiently expressed in tobacco leaves alone. 35S-St4CL5-GFP was the transient expression of St4CL5 and GFP fusion protein in tobacco leaves, and the subcellular location of St4CL5 protein was tested. Bars = 20 µm.

Different from other St4CL family genes, which mostly showed positive responses under abiotic stress, the expression of *St4CL5* was significantly decreased in white light, UV, PEG, and ABA treatments. This illustrated that the St4CL5 might distinguish with other St4CL gene family members. Subsequently, we performed subcellular localization experiments of St4CL5 (Figure 9). The results showed that St4CL5 protein was mainly located at the plasma membrane, which was the same as the result of subcellular localization prediction (Table 1). However, the St4CL5 protein was also distributed in the cytosol, which may be one of the reasons why St4CL5 exhibits differences from other St4CLs.

## 3. Discussion

A total of 10 St4CL family genes were identified in this study in the potato genome. Both AMP-binding and AMP-binding_C conserved domains were contained in potato and Arabidopsis protein by comparative analysis with the Arabidopsis 4CL gene family. These two conserved domains were amino acid sequences that were necessary for *4CL* genes in different plants to perform biological functions [24]. In parallel, 4CL family genes are present in both monocotyledons and dicotyledons genomes [40]. The research showed that the function of 4CL was indispensable in plants, and it was an indispensable coenzyme A ligase in the synthesis of secondary metabolites such as plant lignin [41].

Phylogenetic analysis and gene structure analysis are important means to study the process of gene family evolution [42]. The evolutionary study of the 4CL gene family revealed that in different plants the *4CL* genes were universally divided into two subgroups, one type that was generally associated with flavonoid biosynthesis and the other with lignin and other phenylpropanoids biosynthesis [41]. In total, four 4CL family genes had been identified in Arabidopsis, among which At4CL1, At4CL2, and At4CL4 were mainly involved in lignin biosynthesis and At4CL3 was mainly responsible for flavonoid biosynthesis [4]. The 4CL gene family in the monocotyledonous plant rice could similarly be divided into two classes, Os4CL1, Os4CL3, Os4CL4, and Os4CL5, which were responsible for catalyzing the formation of lignin from hydroxycinnamic acid derivatives, and Os4CL2 after being activated by ultraviolet was mainly specifically expressed in anthers and was involved in flavonoids synthesis [20]. The Potato 4CL family genes also exhibited similar characteristics. Phylogenetic analysis showed that there are two branches of the 4CL gene family in potato, subgroup A containing St4CL1, St4CL2, St4CL3, St4CL4, St4C5, St4CL8, and subgroup B containing St4CL6, St4CL7, St4CL9, St4CL10 (Figure 2). From the results of gene structure analysis, the number of exons of genes in subgroup B was significantly more than that in subgroup A (Figure 3a). The above results illustrate that the St4CL gene family, similar to Arabidopsis and rice, can be divided into two subgroups, and that the genes in different 4CL subgroups differ in gene structure.

However, motif analysis and tandem replication analysis established a connection between subgroup A and subgroup B of St4CL gene family in potatoes [43]. Correlations in the amino acid sequence also existed between subgroup A and subgroup B of the St4CL gene family [44]. St4CLs contained in subgroup B that did not differentiate from other plant 4CLs all contained motif 6, and only 4CL1 contained motif 6 in subgroup A (Figure 3b). In subgroup A, *St4CL1* was a tandem duplication gene with *St4CL2*, but St4CL2 was devoid of motif 6 (Figure 4). This illustrated that the loss of motif 6 occurred during this gene tandem duplication event, possibly leading from here on to the gradual evolution of St4CL gene family into two types, subgroup A and subgroup B. Therefore, motif 6 was also the link between subgroup A and subgroup B in gene structure and phylogeny.

Through comparative genomic analysis of Arabidopsis, rice, tomato, and potato, the collinearity of 4CL gene family between monocotyledons and dicotyledons was studied. The results showed that potato *St4CL6* was simultaneously collinearity with 4CL gene family in Arabidopsis, tomato, and rice. Therefore, *St4CL6* may play an important role in the process of gene expansion of 4CL gene family in potato genome. *St4CL6* has collinearity with Arabidopsis *At4CL3* and rice *Os4CL2*, respectively. The research showed that both *At4CL3* and *Os4CL2* are mainly responsible for the biosynthesis of flavonoids in plants, so it is inferred that *St4CL6* also plays an important role in the biosynthesis of potato flavonoids [9,45]. *St4CL7* and *St4CL10* in the potato were likewise collinearity with *Sl4CL3* and *Sl4CL6* in the tomato, which illustrated the close evolutionary relationship of the 4CL gene family in potato and tomato genomes (Figure 5c). The research showed that the gene expression of tomato *Sl4CL3* and *Sl4CL6* increased significantly under high nitrogen concentration stress, which implied that *St4CLs* might be involved in potato responses to multiple abiotic stresses.

After analyzing the promoters of St4CL family genes, they were found to contain light responsive elements, ABRE cis-acting elements in response to ABA signaling and PEG simulated drought, and MeJA cis-acting elements in response to MeJA treatment [46,47]. The cis-acting elements in the promoters act as molecular switches to regulate gene expression; therefore, the expression of *St4CLs* may be affected by abiotic stresses such as light, ABA, MeJA, and drought treatments [48]. Combining RNA-seq with RT-qPCR results further confirmed that *St4CL* genes were involved in the response to multiple abiotic stresses. Based on the comprehensive analysis of the expression of St4CL family genes under different treatments, it similarly could be found that under white light, UV, PEG, ABA and MeJA treatments, 70% of the St4CL family genes have significantly increased or remained unchanged (Figure 8). Studies of *4CLs* in *Capparis spinosa* have shown that treatments with MeJA and salicylic acid (SA) produced significant changes in *4CL* gene expression [49]. The research of *4CL* genes in *Coleus forskohlii* further illustrated that up-regulated expression of *4CLs* could be strongly induced by ABA treatment [50]. Thus, the biological functions undertaken by the 4CL gene family in plants are not only key enzymes involved in the biosynthesis of secondary metabolites, but also key genes in plants responding to abiotic stress, which are deeply involved in the expression regulatory network of plants for abiotic stress [51].

Both photo-stress caused by UV irradiation and PEG to simulate drought stress can lead to massive accumulation of reactive oxygen species (ROS) in plant cells [52]. ABA treatment and MeJA treatment also induce the production of large amounts of ROS in plant cells [53]. It is well known that excessive accumulation of ROS is an important reason that causes plants to suffer damage under abiotic stress [54]. Research of *4CLs* in rice and Arabidopsis revealed that *Os4CL2* and *At4CL3*, whose expression was significantly upregulated upon UV irradiation, were mainly involved in flavonoids biosynthesis [4,20]. Flavonoids such as anthocyanins are highly capable of scavenging ROS in plants [55]. *St4CL6* also showed collinearity with both *Os4CL2* and *At4CL3*, and gene expression of *St4CL6* was highly increased under abiotic stresses such as UV, PEG, ABA, and MeJA in potatoes. It is therefore speculated that the functions of the genes of St4CL subgroup B represented by *St4CL6* may be similar to *Os4CL2* and *At4CL3*, which promote the accumulation of flavonoids by increasing gene expression to improve the ability to scavenge ROS, and then increase the ability of potatoes to tolerate abiotic stresses [56]. However, in contrast to *St4CL* subfamily B, *St4CL8* in St4CL subgroup A was also significantly up-regulated under multiple abiotic stresses. The research of *Fraxinus mandshurica* 4-coumarate: CoA ligase (Fm4CL2) showed that phenylalanine ammonia-lyase 1 (PAL1) gene expression was greatly increased in transgenic tobacco of overexpression *Fm4CL2* (Fm4CL2oe) under abiotic stress, which increased coniferyl alcohol content in Fm4CL2oe and then led to higher content of lignin, thereby improving the ability of Fm4CL2oe to scavenge ROS to increase abiotic stress tolerance [57]. Possibly different from subgroup B, the biological function of subgroup A containing *St4CL8* gene may be like that of *Fm4CL2*, improving the ability of potato to reduce ROS by promoting the accumulation of lignin, and then improving potato abiotic stress tolerance in potatoes [58].

There were also genes with significantly decreased expression in the potato 4CL gene family under abiotic stress. *St4CL5* in subgroup B showed a significant decrease in gene expression when treated with white light, UV, PEG and ABA, implying that St4CL subgroup A and subgroup B may be functionally differentiated despite being identical 4CL family. In this study, the St4CL5 subcellular localization assay was performed. The 4CL proteins are predominantly distributed in bio-membrane system of plant cell [59], but *Peucedanum praeruptorum* Dunn Pp4CL1 and Pp4CL10, which have different functions compared to other Pp4CL family genes, are localized to the cytosol [60]. Recent results of subcellular localization of Cs4CL1 and Cs4CL2 in *Camellia sinensis* have shown that Cs4CL1 and Cs4CL2 were also mainly distributed in the cytosol [8]. The present study showed that St4CL5 protein was distributed on the plasma membrane, but St4CL5 protein also had an amount of distribution in the cytosol. This is similar to the findings for 4CL gene family proteins in *Peucedanum praeruptorum* Dunn and *Camellia sinensis*, where a different subcellular localization of St4CL5 suggests that St4CL5 may have some differences with other potato 4CL genes [61].

## 4. Materials and Methods

### 4.1. Identification of 4CL Gene Family Members in Potato

To identify potato 4CL family genes, protein sequences corresponding to potato 4CL (NP_001305568.1) and Arabidopsis 4CL (AT1G51680) that had been cloned were applied as query sequences [26,62]. We applied blast-2.12.0+ software (https://ftp.ncbi.nlm.nih.gov/blast/executables/blast+/2.12.0/) to perform a local Blast (accessed on 15 March 2022) search of DM_1-3_516_R44_potato. v6.1.hc_gene_models.pep.fa data and an E-value ≤ 1 × e^−10^ as a threshold [28,29]. The models of 4CL gene family containing AMP-binding enzyme domain (AMP-binding; PF00501) and AMP-binding enzyme C-terminal domain (AMP-binding_C; PF13193), downloaded from the Pfam database (http://pfam.xfam.org/ (accessed on 19 March 2022)), was then applied to HMMER software to detect potato protein sequences obtained through Blast. The protein sequences of the St4CL gene family that might be obtained by the above detection were searched in the SMART database (http://smart.embl-heidelberg.de/ (accessed on 22 March 2022)) to further determine that they contained both AMP-binding and AMP-binding_C domains, combined with the KEGG (https://www.kegg.jp/ (accessed on 24 March 2022)) gene annotation results to finally confirm the St4CL gene family contained genes. Then, ExPASy-ProtParam (https://web.expasy.org/protparam/ (accessed on 27 March 2022)) was applied to predict the physical and chemical properties of the obtained potato 4CL protein sequence. The subcellular localization of the St4CL gene family was predicted through BUSCA website (http://busca.biocomp.unibo.it/ (accessed on 30 March 2022)).

### 4.2. Multiple Sequence Alignment and Construction of the Phylogenetic Tree

DNAMAN software was applied to multiple sequence alignment of the protein sequences of the 4CL gene family in potato and Arabidopsis to detect the conserved amino acid sequences of the functional domains. SOPMA website (https://web.expasy.org/protparam/ (accessed on 6 April 2022)) was applied to predict the secondary structures of AMP-binding_C domain in potato and Arabidopsis. The 4CL gene family amino acid sequences in potato, Arabidopsis, maize, rice, tobacco, cotton, mulberry, and wheat (Supplementary Table S1) were aligned by the ClustalW method [1,9,63,64,65,66,67]. The phylogenetic trees were constructed by the Neighbor-Joining (NJ) method using MEGA10 software with a bootstrap test of 1000 replicates [68].

### 4.3. Gene Structure Analysis and Motif Detection of St4CL Gene Family

The gene structures of St4CL gene family members were analyzed through GSDS 2.0 (http://gsds.gao-lab.org/ (accessed on 9 April 2022)). The conserved motifs of *St4CLs* were analyzed using the MEME website (https://meme-suite.org/meme/ (accessed on 11 April 2022)). We determined that the maximum number of motifs was 6, the minimum motif width was 20, and the maximum motif width was 50. In this study, TBtools software was applied to visualize the above results [69].

### 4.4. Tandem Gene Detection and Gene Collinearity Analysis of St4CL Gene Family

The genomic annotation file corresponding to potato genomic data was downloaded at the PCSC website (http://spuddb.uga.edu/ (accessed on 15 April 2022)) to analyze the distribution of *St4CLs* on potato chromosomes. In this research, the criteria for determining whether there were tandem duplication genes in the St4CL gene family were that the sequence similarity exceeded 70%, the interval between the two genes was within five genes, and the distance between the two genes was less than 100 kb [30]. We considered two genes that simultaneously satisfied the above criteria to be tandem genes. The genome annotation files of rice, tomato, and Arabidopsis were downloaded from the Phytozome v13 database (http://spuddb.uga.edu/ (accessed on 19 April 2022)). Then, the collinearity of the 4CL gene family between potato, rice, tomato, and Arabidopsis was analyzed by SPDE 2.0 [70].

### 4.5. Analysis of Cis-Acting Elements in the Promoters of St4CL Gene Family

To investigate the cis-acting elements contained by the St4CL gene promoters, the DNA sequences 2000 bp upstream of the start codon of 4CL gene family members were obtained by retrieving potato genomic data [71]. The promoter sequences of *St4CL* genes were submitted to PlantCARE (http://bioinformatics.psb.ugent.be/webtools/plantcare/html/ (accessed on 25 April 2022)) predicted cis-acting elements on the promoter.

### 4.6. St4CL Genes Tissue Expression Analysis and Protein Interaction Network Analysis

The potato transcriptomics sequencing data were downloaded from PGSC (http://spuddb.uga.edu/ (accessed on 28 April 2022)). As an orthogonal validation, all samples were sequenced by transcriptome next-generation sequencing [72]. The TPM values of St4CL gene family members in potatoes were obtained, and the expression of each *St4CL* was calculated by the log2 (TPM + 1) [73]. The heatmaps were drawn by applying the Heml software [74]. The protein sequences of all ten genes in the potato 4CL gene family were searched in the string database (http://cn.string-db.org/ (accessed on 10 May 2022)), and the organisms were set as *Solanum tuberosum*. Microsoft Excel was applied to collate the protein–protein interaction results of each node of St4CL gene family members, and the protein–protein interaction network result diagram of St4CL gene family in potatoes was drawn by Cytoscape software [75].

### 4.7. Plant Material and Abiotic Stress Treatment

The potato cultivar used in this study was Desiree. Potato tissue culture seedlings were grown in Murashige and Skoog (MS) liquid medium, pH 5.8, and containing 3% sucrose. Tissue culture seedlings were grown in MS liquid medium for 4 weeks before use in experiments, and tissue culture seedlings were grown in an incubator at 22 °C, 16 h light (12,000 Lx), 8 h dark, and 60% relative humidity. Potato seedlings with good growth status were subjected to the following abiotic stress treatments: 50 umol/L abscisic acid (ABA), 25 umol/L methyl jasmonate (MeJA), 30% (WT/VOL) polyethylene glycol (PEG4000) to simulate drought stress, and the control was potato seedlings without being treated [76]. We sampled potato seedling roots of treated and control groups for RT-qPCR test at 0 h, 3 h and 24 h of treatment, respectively. In addition, normal grown potato seedlings were subjected to white light treatment and UV irradiation treatment after 48 h of continuous dark treatment, and the control group was potato seedlings that continued to be dark treated [11]. The light intensity for white light treatments was 15,000 Lx. One white light tube (LED-22W), one UV-A light tube (UVA-40W), and one UV-B light tube (UVB-40W) were used for UV treatment of potato seedlings subjected to UV-containing white light irradiation [20]. Potato seedling leaves of control and treated groups were sampled at 0 h, 3 h, and 24 h of white light treatment and UV treatment, respectively. All the above five treatment experiments were performed in three biological replicates.

### 4.8. RT-qPCR Analysis and St4CL5 Subcellular Localization

The primers for St4CL gene family members required for RT-qPCR assays were designed by Primer Premier 5.0 software (Supplementary Table S4). RNAsimple Total RNA Kit (TIANGEN, Beijing, China) was applied to extract total RNA from the potato samples. The cDNA was obtained by reverse transcription assay using FastKing RT Kit With gDNase (TIANGEN, Beijing, China). RT-qPCR was performed using SuperReal PreMix Color SYBR Green (TIANGEN, Beijing, China) with ef1a as reference gene, and RT-qPCR assays were performed with three replicates [29]. All above experiments were performed in strict accordance with the corresponding kit instructions. The instrument used for RT-qPCR in this study was Applied Biosystems Q7 Real-Time PCR Systems (Foster city, CA, USA), and the reaction conditions for RT-qPCR were as follows: pre-incubation at 95 °C for 15 min, then 40 cycles of 95 °C for 15 s, 60 °C for 20 s and 72 °C for 20 s, and with a default procedure for melt curve. The relative expression levels of St4CL family genes were calculated by the 2^−ΔΔCt^ method.

The full-length coding sequence of St4CL5 without stop codon was cloned, isolated, and linked into pGFP vector containing GFP reporter gene (saved in our laboratory). The competent cells of *E. coli* (DH5α) and *Agrobacterium* (LBA4404) were used for transformation of recombinant plasmids. The primers required for gene cloning are in Supplementary Table S4. *Agrobacterium*-mediated transient expression in tobacco leaves was performed, as previously described [71,77]. Tobacco leaves transiently transformed by *Agrobacterium* were photographed using the laser scanning confocal microscopy (Olympus FV3000, Tokyo, Japan).

## 5. Conclusions

In conclusion, 10 St4CL family genes were identified in the potato genome. The gene structure, phylogenetic relationships, and cis-acting elements of gene promoters of the St4CL gene family were analyzed by bioinformatics methods, and then the collinearity of the 4CL gene family among different species was compared. The St4CL gene family was shown to be involved in the biosynthesis of lignin and flavonoids by protein interaction network analysis, while it was also associated with gingerol biosynthesis as well as ubiquinone and other terpenoid-quinone biosynthesis. Both RNA-seq data analysis and RT-qPCR results indicated significant changes in the expression levels of St4CL family genes upon white light, UV, PEG, ABA, and MeJA treatment. Therefore, St4CLs were not only enzymes with the key catalytic role in the biosynthesis of secondary metabolites in potatoes but were also involved at the transcriptional level in regulating potato responses to abiotic stress signals such as UV, ABA, MeJA as well as PEG simulated drought stress. The St4CL gene family is functionally complex, and its involvement in the mechanisms of potato response to different environmental signals still needs to be investigated experimentally in future work.

## Figures and Tables

**Table 1:** 10 potato 4CL family genes were identified and protein properties predicted.

Gene	Gene ID	CDS Length (bp)	No. of Exons	Protein			Subcellular Localization
				Length (aa)	MW (Da)	pI	
St4CL1	Soltu.DM.02G004660	1659	1	552	61,037.47	7.55	Organelle membrane
St4CL2	Soltu.DM.02G004670	1656	1	551	60,841.01	7.29	Plasma membrane
St4CL3	Soltu.DM.02G020860	1728	2	575	64,250.28	8.16	Plasma membrane
St4CL4	Soltu.DM.02G020870	1710	2	570	63,664.47	7.24	Organelle membrane
St4CL5	Soltu.DM.03G003110	1851	3	616	68,009.17	7.14	Plasma membrane
St4CL6	Soltu.DM.03G020790	1710	6	569	61,825.23	5.53	Organelle membrane
St4CL7	Soltu.DM.03G032090	1638	5	545	59,636.17	5.49	Plasma membrane
St4CL8	Soltu.DM.06G000670	1647	2	548	60,436.68	8.21	Organelle membrane
St4CL9	Soltu.DM.06G024540	1647	6	548	60,099.43	5.43	Organelle membrane
St4CL10	Soltu.DM.12G011270	1647	6	548	60,157.6	5.36	Plasma membrane

**Table 2:** The conserved motifs in the St4CL protein sequences.

Motif	Length	Amino Acid Sequence
Motif 1	50	EGWLHTGDJGYIDDDGYLYIVDRLKELIKYKGFQVAPAELEALLLSHPEI
Motif 2	29	SEDDPAALPYSSGTTGLPKGVVLTHRNLV
Motif 3	29	NTMGEICLRGPQIMKGYLNBPEATSKTID
Motif 4	29	DAAVVPMPDEZAGEVPVAFVVRSNGSTJT
Motif 5	23	EDEIIDFIAKQVPPYKRIKRVIF
Motif 6	41	YDLSSLRSVMSGAAPLGKELEEAFRKKFPNAKLGQGYGMTE

## Data Availability

Not applicable.

## Supplementary Materials

The following supporting information can be downloaded at: https://www.mdpi.com/article/10.3390/ijms24021642/s1.
