# Supplementary material for: Genome-Wide Identification and Expression Analysis of the 4-Coumarate: CoA Ligase Gene Family in Solanum tuberosum
Source: Int J Mol Sci. 2023 Jan 13;24(2):1642. doi: 10.3390/ijms24021642 (PMC9866895; doi:10.3390/ijms24021642)
Supplement: Supplementary file 1 [file ijms-24-01642-s001.zip › ijms-2125299-supplementary.pdf]

# Genome-Wide Identification and Expression Analysis of the 4-Coumarate: CoA Ligase Gene Family in *Solanum Tuberosum*

## Supplementary Materials

**Table S1.** Protein sequences that were applied to phylogenetic relationship analysis and collinearity analysis.

| Species | Gene Name | Locus ID |
|---|---|---|
| *Solanum tuberosum* | St4CL1 | Soltu.DM.02G004660 |
| | St4CL2 | Soltu.DM.02G004670 |
| | St4CL3 | Soltu.DM.02G020860 |
| | St4CL4 | Soltu.DM.02G020870 |
| | St4CL5 | Soltu.DM.03G003110 |
| | St4CL6 | Soltu.DM.03G020790 |
| | St4CL7 | Soltu.DM.03G032090 |
| | St4CL8 | Soltu.DM.06G000670 |
| | St4CL9 | Soltu.DM.06G024540 |
| | St4CL10 | Soltu.DM.12G011270 |
| *Arabidopsis thaliana* | At4CL1 | AT1G51680 |
| | At4CL2 | AT3G21240 |
| | At4CL3 | AT1G65060 |
| | At4CL4 | AT3G21230 |
| *Oryza sativa* | *Os4CL1* | LOC_Os08g14760 |
| | *Os4CL2* | LOC_Os02g46970 |
| | *Os4CL3* | LOC_Os02g08100 |
| | *Os4CL4* | LOC_Os06g44620 |
| | *Os4CL5* | LOC_Os08g34790 |
| *Zea mays* | Zm4CL1 | AQK68971.1 |
| | Zm4CL2 | XP_008678982.1 |
| | Zm4CL3 | PWZ20321.1 |
| | Zm4CL4 | PWZ07493.1 |
| | Zm4CL5 | NP_001352435.1 |
| | Zm4CL6 | ONM00377.1 |
| | Zm4CL7 | ONL93738.1 |
| *Morus notabilis* | Mn4CL1 | EXB80937.1 |

| Species | Gene Name | Locus ID |
| --- | --- | --- |
| *Morus notabilis* | Mn4CL2 | XP_010092099.1 |
| | Mn4CL3 | XP_010087481.1 |
| | Mn4CL4 | EXC27894.1 |
| *Nicotiana tabacum* | Nt4CL1 | NP_001312667.1 |
| | Nt4CL2 | NP_001312554.1 |
| | Nt4CL3 | XP_016496866.1 |
| | Nt4CL4 | XP_016470276.1 |
| | Nt4CL5 | XP_016466127.1 |
| | Nt4CL6 | XP_016509143.1 |
| | Nt4CL7 | XP_016475356.1 |
| *Triticum aestivum* | Ta4CL1 | XP_044376362.1 |
| | Ta4CL2 | XP_044420438.1 |
| | Ta4CL3 | XP_044408266.1 |
| | Ta4CL4 | XP_044383899.1 |
| | Ta4CL5 | XP_044455635.1 |
| *Gossypium hirsutum* | Gh4CL1 | QOE83936.1 |
| | Gh4CL2 | QOE83937.1 |
| | Gh4CL3 | QOE83938.1 |
| | Gh4CL4 | QOE83939.1 |
| | Gh4CL5 | QOE83940.1 |
| | Gh4CL6 | QOE83941.1 |
| | Gh4CL7 | QOE83942.1 |
| | Gh4CL8 | QOE83943.1 |
| | Gh4CL9 | QOE83944.1 |
| | Gh4CL10 | QOE83945.1 |
| | Gh4CL11 | QOE83946.1 |
| | Gh4CL12 | QOE83947.1 |
| | Gh4CL13 | QOE83948.1 |
| | Gh4CL14 | QOE83949.1 |
| | Gh4CL15 | QOE83950.1 |
| | Gh4CL16 | QOE83951.1 |
| | Gh4CL17 | QOE83952.1 |
| | Gh4CL18 | QOE83953.1 |
| | Gh4CL19 | QOE83954.1 |
| | Gh4CL20 | QOE83955.1 |
| | Gh4CL21 | QOE83956.1 |
| | Gh4CL22 | QOE83957.1 |
| | Gh4CL23 | QOE83958.1 |
| | Gh4CL24 | QOE83959.1 |
| | Gh4CL25 | QOE83960.1 |
| | Gh4CL26 | QOE83961.1 |
| *Gossypium hirsutum* | Gh4CL27 | QOE83962.1 |

| Species | Gene Name | Locus ID |
|---|---|---|
| | Gh4CL28 | QOE83963.1 |
| | Gh4CL29 | QOE83964.1 |
| | Gh4CL30 | QOE83965.1 |
| | Gh4CL31 | QOE83966.1 |
| | Gh4CL32 | QOE83967.1 |
| | Gh4CL33 | QOE83968.1 |
| | Gh4CL34 | QOE83969.1 |
| *Solanum lycopersicum* | Sl4CL1 | Solyc03g097030 |
| | Sl4CL2 | Solyc03g117870 |
| | Sl4CL3 | Solyc06g068650 |
| | Sl4CL4 | Solyc08g076300 |
| | Sl4CL5 | Solyc11g069050 |
| | Sl4CL6 | Solyc12g042460 |

**Table S2.** The transcriptome sequencing TPM values were calculated to obtain the St4CL genes expression of different tissues.

| Gene | Shoots | Petioles | Leaves | Sepals | Carpels | Petals | Stamens | Flowers | Stolons | Tubers | Roots | Callus |
|---|---|---|---|---|---|---|---|---|---|---|---|---|
| St4CL1 | 0.56 | 0.00 | 0.05 | 0.21 | 0.13 | 0.21 | 0.00 | 0.13 | 0.00 | 0.00 | 0.11 | 0.00 |
| St4CL2 | 5.15 | 6.49 | 5.97 | 5.66 | 4.03 | 6.86 | 8.19 | 7.07 | 5.11 | 4.07 | 4.85 | 4.66 |
| St4CL3 | 1.40 | 4.38 | 1.16 | 1.14 | 0.14 | 0.00 | 1.10 | 1.45 | 1.33 | 0.25 | 0.12 | 0.91 |
| St4CL4 | 2.04 | 6.05 | 2.13 | 2.08 | 2.29 | 0.68 | 0.57 | 1.51 | 2.84 | 1.66 | 0.95 | 1.20 |
| St4CL5 | 4.65 | 1.22 | 3.42 | 5.13 | 4.62 | 8.09 | 0.64 | 6.41 | 2.51 | 0.13 | 1.51 | 0.97 |
| St4CL6 | 3.41 | 3.69 | 3.91 | 3.85 | 2.72 | 4.09 | 4.73 | 4.24 | 5.84 | 6.34 | 2.99 | 2.30 |
| St4CL7 | 4.25 | 3.32 | 3.18 | 3.51 | 5.43 | 4.81 | 5.72 | 4.62 | 7.65 | 6.54 | 3.96 | 5.57 |
| St4CL8 | 2.44 | 1.69 | 2.61 | 4.22 | 4.52 | 4.40 | 6.58 | 4.29 | 3.11 | 3.45 | 3.46 | 3.46 |
| St4CL9 | 3.90 | 3.85 | 3.57 | 3.88 | 3.85 | 3.07 | 1.30 | 2.14 | 5.65 | 5.23 | 4.16 | 4.61 |
| St4CL10 | 2.19 | 3.33 | 1.51 | 1.91 | 1.19 | 1.06 | 0.67 | 0.22 | 2.50 | 0.65 | 2.84 | 1.11 |

**Table S3.** The transcriptome sequencing TPM values were calculated to obtain the St4CL genes expression of different treatments.

| Gene | *P. infestans* | BABA | BTH | NaCl | Mannitol | Heat | ABA | IAA | GA3 | BAP |
|---|---|---|---|---|---|---|---|---|---|---|
| St4CL1 | 0.00 | -1.22 | -1.11 | 0.09 | -0.52 | -0.07 | -0.98 | -0.30 | -0.34 | -0.69 |
| St4CL2 | -0.27 | 1.48 | 0.10 | -0.08 | 0.29 | 0.72 | 0.66 | 0.09 | 0.31 | -0.40 |
| St4CL3 | -0.97 | 2.46 | 0.94 | 0.04 | -0.06 | -1.08 | 1.36 | 0.86 | 0.58 | 0.84 |
| St4CL4 | -1.35 | 2.57 | -0.34 | -0.16 | 0.34 | -1.49 | 1.02 | 0.17 | 0.12 | -0.12 |
| St4CL5 | 0.25 | -4.10 | -2.46 | 0.38 | 0.24 | -3.78 | -0.78 | 0.07 | 0.22 | -1.67 |
| St4CL6 | -0.70 | 2.17 | 1.63 | 0.08 | 0.34 | -0.21 | 0.79 | 0.16 | 0.06 | -0.13 |
| St4CL7 | -0.98 | 1.65 | -0.68 | -0.24 | 0.01 | 0.86 | 1.20 | 0.06 | 0.58 | -0.30 |
| St4CL8 | -0.09 | -0.23 | 2.32 | -0.36 | -0.34 | 1.98 | 1.57 | 0.78 | 0.02 | 0.07 |
| St4CL9 | -1.31 | -1.02 | -0.78 | 0.05 | -0.04 | 1.49 | 0.63 | -0.09 | 0.46 | -0.50 |
| St4CL10 | -0.66 | 0.29 | -0.31 | 0.66 | 0.78 | 0.20 | 0.58 | -0.52 | -0.19 | -0.07 |

**Table S4.** Primers used for gene cloning and RT-qPCR.

| Primer name | Sequences (5'-3') | Functions |
|---|---|---|
| St4CL5-C-FP | ATGGACATTTTAACACCAACTAG | Gene cloning |
| St4CL5-C-RP | TACAAGTGATTTGGCTTTAT | Gene cloning |
| St4CL1-FP | GTGCTAAGCCCTTAAAAAACCC | RT-qPCR |
| St4CL1-RP | ATACACGACACGACTACCCCTG | RT-qPCR |
| St4CL2-FP | TCCTCCGTCACAGCGAATCTAA | RT-qPCR |
| St4CL2-RP | AACGAAAATACCCCTATGGC | RT-qPCR |
| St4CL3-FP | GTCTACTTATGGTCCCTCCCTA | RT-qPCR |
| St4CL3-RP | TTATTTTATGCTGCTCGTGTGG | RT-qPCR |
| St4CL4-FP | AAACAAGCAAAAGGGAAAAGCC | RT-qPCR |
| St4CL4-RP | CTAAATCTCCGATGACAACGCA | RT-qPCR |
| St4CL5-FP | CGTCTCGATCCACGTACCCTCT | RT-qPCR |
| St4CL5-RP | GTCTCCTTCATCAATAACATTCTCA | RT-qPCR |
| St4CL6-FP | TAGTTCCGCCCCTGGTTCTTGC | RT-qPCR |
| St4CL6-RP | CTACTGGTCCTGCCTCTGTCAT | RT-qPCR |
| St4CL7-FP | GAGTTTCATTCCCGGCCCTGTT | RT-qPCR |
| St4CL7-RP | TGCGTAGTCCTTCACTTTCCCC | RT-qPCR |
| St4CL8-FP | GCGGAGACAAGACGAAGGTG | RT-qPCR |
| St4CL8-RP | ACAAAGAACTGCCCCAGCCATT | RT-qPCR |
| St4CL9-FP | GGTTACATACGGGCGACATTG | RT-qPCR |
| St4CL9-RP | CCTGCTTGCTCGTCTTTCATTG | RT-qPCR |
| St4CL10-FP | TCTGGATTTACCTCTGAACACGA | RT-qPCR |
| St4CL10-RP | TGACCCCTTTTGGTAGCCCCG | RT-qPCR |
| ef1a-FP | GGAAAAGCTTGCCTATGTGG | RT-qPCR |
| ef1a-RP | CTGCTCCTGGCAGTTTCAA | RT-qPCR |
